# Willing and Readiness of LTCF’s to Admit and Manage CRE Patients – a Multi-Center Study

**DOI:** 10.1017/ash.2025.350

**Published:** 2025-09-24

**Authors:** Owen Simwale, Oliver Kunda

## Abstract

**Background:** Carbapenem-Resistant Enterobacterales (CRE’s) are an imminent and growing threat to our healthcare system, especially those in Long Term Care Facilities (LTCF). Most patients with CRE in the US are diagnosed in hospitals but often discharged to LTCF’s. But how willing and prepared are LTCF’s to take and effectively manage CRE patients? We conducted a multi-center survey of 200 LTCF’s across 4 states (CT, CA, PA, TX) to assess their willingness to admit and preparedness to manage CRE patients. **Objective/methods:** A questionnaire was sent to 200 consenting facilities asking about willingness and readiness to manage a CRE patient. We excluded LTAC’s and SNFv which tend to have more exposure to CRE and thus more open to accept CRE patients. Readiness was measured by capacity built around CDC recommendations for CRE management and prevention in LTCF. Survey results were analyzed using SAS inc. **Results:** Of the 200 surveys sent, 168 were completed and returned. Eighteen (18) were excluded for incompleteness or unclear responses. Of the 150 facilities included in the analysis, only 18 (12%) said they have had experience managing a CRE resident and 41(27%) said they would accept CRE patients. Most common reasons for unwillingness to accept CRE patients were lack of private rooms, fear of causing an outbreak and not being prepared to handle such cases. As for readiness to handle CRE patients 71(47%) said they had a CRE policy, 45 (30%) had contingent plans for how to effectively isolate CRE patients, 60 (40%) said they had isolation signage for CRE, 38 (25%) had cleaning and disinfection supplies for CRE, 21 (14%) had contingent plans for surveillance testing if that were needed. A majority 145 (97%) were aware to both notify public health if they have a case and to use an inter-facility form when transferring the patient to another facility. **Conclusion:** Our study, though small in size, highlights how unwilling and unprepared a lot of LTCF’s are to take on CRE patients. We also highlight some of the barriers and gaps in preparedness that can be addressed to build the capacity of LTCF’s to take in and manage CRE residents effectively.

